# Are Students’ Symptoms and Health Complaints Associated with Perceived Stress at University? Perspectives from the United Kingdom and Egypt

**DOI:** 10.3390/ijerph111009981

**Published:** 2014-09-26

**Authors:** Walid El Ansari, Reza Oskrochi, Ghollamreza Haghgoo

**Affiliations:** 1 Faculty of Applied Sciences, University of Gloucestershire, Gloucester GL2 9HW, UK; 2 Faculty of Technology, Design and Environment, Oxford Brookes University, Oxford OX33 1 HX, UK; E-Mails: roskrochi@brookes.ac.uk (R.O.); grhaghgoo2004@yahoo.com (G.H.)

**Keywords:** psychosomatic symptoms, subjective health complaints, university students, UK, Egypt, college health

## Abstract

This cross-sectional survey assessed and compared by country, the levels and correlates of 21 self-reported symptoms/health complaints. We examined the associations between self-reported symptoms and perceived stress. Data was collected from universities in the United Kingdom and Egypt (N = 3706 and 3271 undergraduates, respectively). A self-administered questionnaire assessed a range of self-reported symptoms, perceived stress, sociodemographic (gender, age, marital status, year of study, living arrangements during semester, income sufficiency), lifestyle (tobacco smoking, illicit drug/s use, alcohol consumption frequency), and health variables (subjective health status, health awareness, BMI), along with religiosity, and quality of life. Factor analysis categorized the 21 self-reported symptoms into four components. Correlation analysis and linear regression tested the associations between the self-reported symptoms and stress. Factor analysis of the health symptoms generated four symptom groups for each of the UK and Egypt (psychological; circulatory/breathing; gastrointestinal; and, pains/aches), and factor loadings were quite similar for both countries. Whilst the two samples showed similarities as to the kind of symptoms most frequently reported by students, the Egyptian sample had significantly higher frequency than the UK for every symptom. Frequent complaints (both countries) included difficulties to concentrate, fatigue, headaches, nervousness/anxiety, and back pain (UK) and mood swings (Egypt). Significantly more Egyptian students reported ≥4 symptoms over the past year than the UK. For each of the UK and Egypt, across each of the four symptom groups, there was a stepladder appearance whereby the frequency of symptoms increased with increasing quartiles of perceived stress. Not controlling for other variables, for both countries, there were significant positive correlations between each of the four symptom groups and stress; the highest correlation was for psychological symptoms. After controlling for sex, age country, and other symptom groups, stress was highly and significantly associated with psychological symptoms and also with pain & aches symptoms in both countries. UK students were generally less stressed than their counterparts in Egypt. Age and female gender were also associated with stress; the younger the student was the more likely to suffer from stress. Interactions were not significant. Across both countries, the levels of stress among students and the associations between perceived stress and health complaints suggest the need for a multiple approaches in order to understand the sources of stress; how college students experience stress; and, the coping mechanisms that different students employ to mitigate stress. Interventions aimed at both preventing, treating and caring for students’ distress, and also preventive strategies to help minimize the impact of stressful situations are required. Strategies that address both physical and psychological complaints may be beneficial for this population.

## 1. Introduction

Time at university is enjoyable for most students, but for some, it could be accompanied by emotional challenges, mood disorders, and relationship issues as students develop friendships and intimate relationships, career choices and pursue personal and professional goals [1,2,3]. Students are also exposed to various psychosocial and physical hazards [4], sometimes with decreased connectedness to their families. Evidence further suggests that some types of university social environments (e.g., the social climate of college living groups) could be “high risk” settings in that they support/facilitate complaints of physical symptoms [5]. Unsurprisingly, in Singapore, about 50% of university students reported significant stress [4].

The stress of such situations and others is a risk factor that may negatively impact on quality of life and health [6]. Such stress and its accompanying psychosomatic complaints could also translate into difficulty in making friends, poor self-evaluation of academic performance, and feelings of dropping out of the course [3]. In Japan, students with psychosomatic disorders (e.g., those involving mood and sleep) were at risk of deleterious changes in their dietary, lifestyle and living environments [7]. University students generally report a wide range of symptoms and health complaints that can be broadly characterized into: (1) psychological health complaints; (2) circulatory and breathing symptoms; (3) gastrointestinal symptoms; and, (4) pains and aches.

In terms of the psychological health complaints and symptoms, mental health issues are increasing in severity and number on college campuses [1]. A review of psychological distress among U.S. and Canadian medical students found a high prevalence of depression and anxiety, with levels of psychological distress consistently higher than in the general population and age-matched peers by the later years of training [8]. Likewise, in Saudi Arabia, students had impaired concentration (34%), memory disturbances (41%), and sleeplessness (38.8%) [9]. In the USA, mood disorders were one of the top three concerns for students pursuing psychological counseling [1]; and sleep disorders are commonly seen at campus mental health services [2].

As regards circulatory/breathing complaints at university, somatic and psychic changes due to stress and difficult situations include tachycardia, excessive perspiration, and menstruation disorders [10]. In terms of breathing, in Belfast, between 1972 and 1989, there was more than two-fold rise in the prevalence of diagnosed asthma, and in non-asthmatics the prevalence of dyspnoea also increased [11]. Such surge in dyspnoea in non-asthmatics [11] might herald the stress that college students encounter.

As for pains and aches, arm, neck and shoulder complaints are prevalent in many societies and an economic problem due to sickness absence and health-care costs [12]. In 2003 the 12-month prevalences in the Netherlands were about 31.4% (neck pain), 30.3% (shoulder pain), and 17.5% (wrist/hand pain). Such complaints are common among college students [12], e.g., Nigerian undergraduates had a high prevalence of musculoskeletal pain, where shoulder pain was most common [13]. In Australia, musculoskeletal disorders were a widespread problem for university students [14]; and in the USA, college students reported low back pain [15], and the majority had musculoskeletal discomfort during/after computer use [16]. Likewise, in Saudi Arabia, students complained of headache (17%) and fatigue (24%) [9].

In connection with gastrointestinal symptoms, in China, university students showed a 15.7% prevalence of irritable bowel syndrome where abdominal pain was a common symptom [17]. Equally, Korean college students had a 5.7% prevalence of irritable bowel syndrome [18]; and in Canada, gastrointestinal symptoms were diagnosed in 51.2% of university students [19].

There is mounting evidence for the relationships between stress and such a range of symptoms/health complaints. For instance, in the USA, irritable bowel syndrome among university students was associated with higher frequency of anxiety, greater worry, and neuroticism [20]. Among Malaysian medical students, psychological and psychosomatic symptoms (anxiety, depression, insomnia, headache, backache) were more frequent in those with irritable bowel syndrome [21]. In China, students exhibited a high prevalence of musculoskeletal symptoms that was associated with psychological distress [22]; and in the USA, higher levels of physical symptoms were associated with psychological distress [23]. Equally, among undergraduates from Spain, Germany, and Lithuania, psychosocial stress was independently and consistently associated with all three (psychosomatic, gastrointestinal, neck ache/backache) complaints [24].

The literature reveals several gaps. First, whereas studies have documented the prevalence of various health complaints, less research undertook the extra step of exploring the relationships between such health complaints/self-reported symptoms and the stress perceived by students during their college years. Secondly, few studies have investigated in detail the relationships between perceived stress and a wide range of self-reported symptoms across large samples of students from different faculties within a variety of universities across different countries. Thirdly, little research has examined the same question (associations between health complaints and perceived stress levels) across different countries employing the same research instruments so that cross-country findings are directly comparable. This is despite that studies among university students that measured their stress levels and compared the findings with those from other countries have highlighted the possibility that any differences observed could be due to the different instruments that different studies employed [25]. Fourthly, the few studies that undertook cross-country comparisons of the differences in health complaints among university students and their relationships with psychosocial stress were mostly limited to European countries e.g., university students from three European countries (Spain, Germany, Lithuania) [24]. To the best of our knowledge, very few studies undertook comparisons between e.g., European and African/Eastern Mediterranean countries with different social contexts, political traditions, and cultures. This is notwithstanding the calls for the need of large, multicenter studies to ascertain features that influence depression and anxiety among students and explore relationships between distress and competency [8]. Others have also called for the need to expand comparative health analysis in terms of the range of countries examined [26]. We selected the UK and Egypt for comparison because whilst the numbers of students are quite similar *i.e.*, ≈2.5 million students registered at 163 higher education institutions in the UK (2011–2012); and ≈2.4 million Egyptians were in post-secondary institute/university (2009–2010), however there are differences between the UK and Egypt. Mainly, the two countries: (1) represent different social environments and settings, political traditions and policy making structures, religions (e.g., UK—mainly Christian; Egypt—mainly Muslims), and cultures (e.g., more individualistic cultures in the UK *vs.* more collectivist cultures in Egypt); (2) represent different educational systems and higher educational contexts; (3) in addition, in Egypt the nature of study in higher education institutions is such that it is customary that most pupils usually proceed from high school directly to university (as males still also need to complete compulsory military service after graduating from university which lasts for a few years). This results in students who are traditionally aged (*i.e.*, a significant fraction of students at universities in Egypt are “fresh” from high school, and hence are relatively younger). In contrast, in the UK or in Europe generally, a sizeable proportion of students graduating from high school might tend take a “break” for a year or two (e.g., to travel overseas) before moving on to university study. This results in students who are relatively older when they start their college studies.

The current study bridges these knowledge gaps to examine the relationships between perceived stress and a wide range of self-reported symptoms across large samples of students from different faculties within a variety of universities across a European (United Kingdom) country and an Eastern Mediterranean (Egypt) country. These features collectively attach high importance to the findings of the present study.

### Aim of the Study

We undertook a cross-sectional survey of a representative sample of students across universities in the UK and in Egypt. Data was collected during the academic years 2007–2008 (UK) and 2009–2010 (Egypt). The study assessed the relationships between perceived stress and many self reported symptoms/health complaints for each of the two samples, and compared the findings across the UK and Egypt. The four specific objectives were to:

- Describe and compare the general characteristics of the two samples; and undertake factor analysis of 21 self-reported symptoms/health complaints into appropriate components;
- Assess and compare the prevalence of 21 symptoms/health complaints and the number of symptoms/health complaints reported in the last 12 months;
- Explore and compare the frequency of symptoms/health complaints by perceived stress level; and,
- Compare the association between symptoms and perceived stress without controlling for any variables; and with controlling for sex and age (presented for each country separately, and both countries combined, and also controlled for other symptoms).

## 2. Materials and Methods

The scope of the current research is in line with other general student health and wellbeing surveys undertaken in a number of countries [27,28,29,30,31,32,33,34,35,36,37].

### 2.1. Ethics and Data Collection

For both countries, the university ethics committees at the participating universities ethically approved the study. Selection of the universities was premised on research interests, existing contacts and history of previous successful collaboration. In both the UK and Egypt, participants were informed that by completing the questionnaire, they consent to participate in the study, and data were confidential/protected. Students attending regular lectures of randomly selected courses at the universities completed self-administered questionnaires during the last 10–15 min of their classes. For quality assurance, all data were computer entered by one person in the UK and one person in Egypt to minimize data entry errors. No monetary or course credit incentives were provided to students for participation.

In Egypt, data were collected (academic year 2009/2010) from a representative random sample of students at Assiut University (≈10% of students) at each of the eleven participating faculties: Business (N = 604, 18.8%), Engineering (N = 572, 17,8%), Education (N = 461, 14.4%), Arts (N = 424, 13.2%), Social Work (N = 328, 10.2%), Sciences (N = 206, 6.4%), Physical Education (N = 178, 5.5%), Computers & Information (N = 137, 4.3%), Veterinary Medicine (N = 131, 4.1%), Specific Education (N = 119, 3.7%), and, Agriculture (N = 50, 1.6%). The Egyptian data comprised 3,271 students (47.5% males, 52.5% females; mean age 18.9 ± 1.4 years). Based on the number of completed and returned questionnaires, the response rates were about ≈90%.

In the UK, data was collected (academic year 2007/2008) simultaneously at seven universities in three countries of the UK: England, University of Gloucestershire (N = 970, 26.2% of the sample); Bath Spa University (N = 485, 13.1%); Oxford Brookes University (N = 208, 5.6%); University of Chester (N = 993, 26.8%); Plymouth University (N = 169, 4.6%); Wales, Swansea University (N = 406, 11.0%); and the Republic of Northern Ireland, University of Ulster (N = 475, 12.8%). The data employed in the current analysis comprised 3,706 students (765 males, 2,699 females, 242 missing gender; mean age 24.9 ± 8.6 years). Based on the number of returned questionnaires, the response rates were ≈80%.

### 2.2. Student Health & Wellbeing Questionnaire

This study utilized a self-administered questionnaire that included general health and wellbeing information, as well as the Perceived Stress Scale and 21 symptoms. The self-reported information included sociodemographic features (gender, age, marital status, year of study, living arrangements during university time, income sufficiency), lifestyle characteristics (tobacco use—smoking, illicit drug/s use, alcohol consumption frequency), health variables [subjective health status (self-rated health), health awareness, height and weight (to compute BMI)], religiosity (importance of religion/personal faith), and quality of life.

Perceived Stress Scale (four items): We employed Cohen’s Perceived Stress Scale (PSS) in its four item short form [38] to measure the extent to which participants considered life situations to be stressful. PSS-4 is a simple psychological instrument that assesses the degree to which situations in one’s life over the past month are considered as stressful. These are general questions designed to detect how unpredictable, uncontrollable, and overloaded respondents find their lives. All items began with the same phrase: “In the past month, how often have you felt…?” (5-point scale: 0 = “never”, 1 = “almost never”, 2 = “sometimes”, 3 = “fairly often”, 4 = “very often”). Higher scores indicated more perceived stress. In the current study, Cronbach’s alpha of the PSS was 0.591and 0.455 (UK and Egypt samples respectively).

Health problems, strains and psychosomatic symptoms (21 items): participants rated 21 symptoms measuring a raft of health complaints as adopted from Stock *et al.* [24,39]. Sample items included stomach trouble/heartburn, back pain, rapid heartbeats/circulatory problem/dizziness, headaches, sleep disorder/insomnia, concentration difficulties, neck and shoulder pain, and depressive mood. Respondents rated the question: “How often have you had these complaints during the past 12 months?” (four-point response scale, 1 = “never”; 4 = “very often”). In the current study, Cronbach’s alpha of the whole scale (21 items) was 0.885 and 0.877 (UK and Egypt samples respectively).

#### Sociodemographic Variables

Age (one item): “What is your age?” Participants reported their age in years. We then categorized age into three categories (≤20 years, 20 to ≤24 years, >24 years).

Year of study (one item): “What is your current year of study at university?” response options included “1st year”, “2nd year”, “3rd year”, and “4th year or above”.

Marital status (1 item): “What is your marital status?” with four options: “single”, “married”, “in a relationship”, “other”. These responses were then collapsed into two groups (“single”, “not single”).

Living arrangements during university semester time (one item): “Where do you live (during university/college term time)?” with four response options (“I live alone”, “I live together with my partner”, “I live with my parents”, “I live with room mates”). For the analysis, these were collapsed into two categories (“living with parents”, “not living with parents”).

Income sufficiency (subjective economic position) (one item): participants rated how sufficient or insufficient they considered the amount of money they have at their disposal is (4-point scale: “always sufficient”, “mostly sufficient”, “mostly insufficient”, “always insufficient”), later collapsed into two groups (“always/mostly sufficient”, “always/mostly insufficient”).

#### Lifestyle Characteristics

Tobacco smoking (one item): “Within the last 3 months, how often did you smoke? (cigarettes, pipes, cigarillos, cigars)” rated on a 3-point response scale (“daily”, “occasionally”, “never”).

Illicit drug/s use (one item): “Have you ever used drug/s?” (“yes, regularly”, “yes, but only a few times”, “never”).

Frequency of alcohol consumption (one item): “Over the past three months how often have you drunk alcohol, for example, beer?” (“never”, “once a week or less”, “once a week”, “a few times each week”, “every day”, “a few times each day”).

#### Health Variables

Subjective health status (one item): “How would you rate your health in general?” (5-point scale response: 1 = “excellent”, 2 = “very good”, 3 = “good”, 4 = “fair”, 5 = “poor”) as employed in the German Federal Health Survey [40] (similar wording used by American College Health Association) [41].

Health awareness (one item): students were asked about their general awareness (surveillance) of their health “To what extent do you keep an eye on your health?” (4-point response: 1 = “not at all”, and 4 = “very much”) [24].

BMI: calculated from self-reported weight and height using Metric BMI Formula—BMI (kg/m^2^) = weight in kilogram/the squared height (m^2^). Using World Health Organization guidelines [42], BMI was then grouped into: underweight (BMI < 18.5 kg/m^2^), normal (18.5 ≤ BMI ≤ 24.9 kg/m^2^), overweight (25.0 ≤ BMI ≤ 29.9 kg/m^2^), or obese (BMI ≥ 30.0 kg/m^2^).

#### Religiosity

Importance of religion/personal faith (one item): participants rated the extent to which they agreed with the following statement: “My religion is very important for my life” (5-point response options: 1 = “strongly agree” to 5 = “strongly disagree”).

#### Quality of Life

Quality of life (one item): “If you consider the quality of your life: How did things go for you in the last four weeks?” based on the quality of life measurement charts [43] with 5 response categories (“poor”, “fair”, “good”, “very good”, “excellent”). The variable was further re-coded into three new categories: “poor/fair”, “good”, and “very good/excellent” quality of life.

### 2.3. Statistical Analysis

We used the Statistical Package SPSS v19.0 for the statistical analyses. For each of the UK and Egypt samples, we employed factor analysis (using principal component analysis, PCA) to determine the number and composition of underlying dimensions to be used in subsequent analyses, by merging the original 21 questionnaire items (symptoms/health complaints) into meaningful constructs. Two points suggested that data was suitable for factor analysis: the sample size of 3706 (UK) and 3271 (Egypt) questionnaires was more than the minimum 300 recommended for PCA; and the ratio of questionnaires to items was about 168 to 1, higher than the suggested 10 to 1 ratio. We evaluated the data for suitability for PCA, and a scree plot of the eigenvalues was employed to decide on the number of components, as this is better than retaining all the dimensions with eigenvalues >1. In factor analysis and PCA, each component is constructed as a weighted average of the questions that comprise it (factor scores); *i.e.*, each component can be regarded as scale measure of the collective severity of those questions that comprise the component. Hence, a component value close to 1 corresponds to “Never” (or no severity), whereas a component value close to 4 corresponds to “Very often”.

For internal consistency, reliability analyses using Cronbach’s Alpha were undertaken on the items that comprised each of the four components (groups of symptoms) that emerged from the factor analysis. We measured the correlation between the four components using Pearson coefficient that emerged from the factor analysis.

For the descriptive analysis, categorical data were expressed as frequencies and percentages, and score data as means and standard deviations. Perceived stress was categorized into four levels based on quartiles. The first quartile (Q1) represented the lowest quarter of perceived stress level; the fourth quartile (Q4) represented the highest quarter of perceived stress level. ANOVA tests examined the differences among the quartiles of perceived stress (Q1‒Q4) in relation to the students’ reported severity of symptoms within and between countries.

Correlation analysis initially assessed the association between perceived stress level and each of the four groups of symptoms within and between countries. Then we undertook several multiple linear regression models, where analyses were conducted while adjusting for the effect of all other groups of symptoms, age, sex and their interactions. In addition, stepwise multiple linear regression analyses were conducted to explore significant effects of the symptoms, other students’ general characteristics and their interactions.

## 3. Results

### 3.1. General Characteristics of the Samples

Table 1 shows that for the sociodemographic features, there were relatively more males in the Egyptian sample than the UK one; and relatively more females in the UK sample compared with Egypt. In Egypt, the sample comprised more of the younger students than the UK, which had relatively more of the older students. Despite this, there were relatively more 1st and 2nd year students in the UK than Egypt. More single persons were in Egypt than the UK, but in the UK, there was relatively more students not living with their parents. More UK students felt they always or mostly had insufficient money. As for the lifestyle characteristics, relatively more students never smoked, never used illicit drug/s, and never used alcohol in Egypt compared to the UK. In connection with the health variables, whilst relatively more UK students reported excellent health and health awareness, there were more students in Egypt with normal BMI. Finally, relatively more Egyptians strongly agreed that religiosity is important in their life, in contrast with the UK where more students strongly disagreed to this statement. For quality of life, relatively more UK participants felt very good/excellent quality of life compared to Egypt.

**Table 1:** General characteristics of the United Kingdom and Egypt samples.

Variables		UK	Egypt	*p*
		N (%)	N (%)	
**Sociodemographic**				
**Gender**	Male	765 (22.10)	1504 (47.50)	<0.001
	Female	2699 (77.90)	1663 (52.50)	
**Age (years)**	≤20	1098 (30.90)	2800 (85.60)	<0.001
	20 < age ≤ 24	855 (24.10)	407 (12.40)	
	>24	1601 (45.00)	64 (2.00)	
**Marital status**	Single	2051 (55.30)	3145 (96.10)	<0.001
	Not single	1655 (44.70)	126 (3.90)	
**Year of study**	1st	1491 (42.60)	1080 (33.60)	<0.001
	2nd	1095 (31.30)	918 (28.50)	
	3rd	655 (18.70)	862 (26.80)	
	≥4th	262 (7.50)	357 (11.10)	
**Living with parents**	No	2816 (76.00)	1978 (61.90)	<0.001
	Yes	890 (24.00)	1218 (38.10)	
**Income sufficiency**	Always/mostly insufficient	1925 (57.50)	712 (22.40)	<0.001
	Always/mostly sufficient	1423 (42.50)	2460 (77.60)	
**Lifestyle**				
**Smoking**	Never	2546 (72.30)	2850 (91.20)	<0.001
	Occasionally	421 (12.00)	174 (5.60)	
	Daily	555 (15.80)	100 (3.20)	
**Illicit drug/s use**	Never	2430 (69.70)	2687 (95.50)	<0.001
	Yes, only a few times	886 (25.40)	115 (4.10)	
	Yes, regularly	168 (4.80)	11 (0.40)	
**Alcohol consumption frequency**	Never	285 (8.10)	2520 (89.60)	<0.001
	≤Once a week	1737 (49.20)	185 (6.60)	
	Several times a week	1325 (37.50)	84 (3.00)	
	Every day/Several times a day	183 (5.20)	23 (0.80)	
**Health**				
**Subjective health status**	Excellent/Very good	1706 (47.50)	597 (18.40)	<0.001
	Good	1503 (41.80)	1531 (47.20)	
	Fair/Poor	385 (10.70)	1114 (34.40)	
**Health Awareness**	Not at all/Not much	633 (17.70)	817 (25.30)	<0.001
	Very much/To some extent	2951 (82.30)	2414 (74.70)	
**BMI (reported)**	Underweight	28 (4.20)	199 (6.10)	<0.001
	Normal	367 (54.90)	2053 (62.80)	
	Overweight	153 (22.90)	709 (21.70)	
	Obese	120 (18.00)	310 (9.50)	
**Religiosity**				
**Importance of religion**	Strongly/somewhat agree	864 (25.30)	3169 (97.70)	<0.001
	Neither agree nor disagree	913 (26.70)	13 (0.40)	
	Strongly/somewhat disagree	1643 (48.00)	63 (1.90)	
**Quality of life**				
**Quality of life**	Poor/fair	274 (7.70)	427 (13.20)	<0.001
	Good	985 (27.70)	1566 (48.50)	
	Very good/Excellent	2297 (64.60)	1235 (38.30)	

### 3.2. Factor Analysis of 21 Self Reported Symptoms/Health Complaints

Table 2 depicts the results of the factor analysis for the health complaint symptoms for each of the UK and Egypt. The symptoms fitted nicely into a four-factor solution, namely: psychological; circulatory/breathing; gastrointestinal; and pains/aches. For each of the two samples, the numbers of individual symptoms that fitted elegantly into each symptom component generated by the factor analysis were nine items, five items, three items, and four items, respectively. In addition, the factor loadings were quite similar for the UK and Egypt.

**Table 2:** Factor analysis of 21 self-reported symptoms/health complaints into four components for the UK and Egypt.

Variable	United Kingdom				Egypt			
	Component				Component			
	1	2	3	4	1	2	3	4
	Psych	Circ/	GIT	PA	Psych	Circ/	GIT	PA
		Breath				Breath		
	(9 items)	(5 items)	(3 items)	(4 items)	(9 items)	(5 items)	(3 items)	(4 items)
Cronbach’s alpha	0.821	0.686	0.671	0.627	0.770	0.710	0.700	0.596
Nervousness/Anxiety	**0.683**	0158	0.076	0.149	**0.701**	0.229	0.012	0.128
Depressive mood	**0.670**	0.244	0.106	0.171	**0.607**	0.234	0.031	0.076
Mood swings	**0.648**	0.095	0.181	0.235	**0.554**	0.108	0.132	0.213
Fear/Phobia	**0.568**	0.297	0.086	0.091	**0.589**	0.297	0.188	0.028
Difficulties to concentrate	**0.618**	0.096	0.014	0.238	**0.536**	0.025	0.037	0.317
Sleep disorder/Insomnia	**0.486**	0.182	0.126	0.301	**0.534**	0.063	0.022	0.271
Nightmares	**0.534**	0.187	0.075	0.171	**0.551**	0.184	0.081	−0.027
Lack of appetite	**0.545**	0.144	0.070	−0.046	**0.485**	0.141	0.082	0.189
Weight gain/Weight loss	**0.553**	0.029	0.257	0.016	**0.442**	0.003	0.165	0.099
Trembling hands	0.167	**0.779**	0.009	0.043	0.072	**0.758**	0.076	0.159
Trembling	0.250	**0.773**	0.043	0.028	0.230	**0.736**	0.106	0.077
Speech impediment	0.145	**0.550**	0.076	0.002	0.274	**0.571**	0.089	−0.192
Breathing difficulties	0.052	**0.439**	0.129	0.318	0.090	**0.568**	0.064	0.342
Rapid heartbeats/Circulatory problems/Dizziness	0.189	**0.556**	0.160	0.239	0.257	**0.472**	0.100	0.321
Diarrhoea	0138	0113	**0.814**	0.050	0.107	0.106	**0.842**	0.108
Constipation	0.168	0.080	**0.804**	0.096	0.098	0.121	**0.838**	0.096
Abdominal problems	0.257	0.175	**0.521**	0.327	0.288	0.121	**0.513**	0.288
Back pain	0.042	0.092	0.070	**0.769**	0.077	0.234	0.123	**0.655**
Neck and shoulder pain	0.171	0.130	0.091	**0.715**	0.135	0.268	0.166	**0.586**
Fatigue	0.324	0.100	0.072	**0.498**	0.314	−0.028	0.085	**0.590**
Headaches	0.365	−0.027	0.097	**0.423**	0.408	−0.052	0.107	**0.490**

Psych: Psychological; Circ/Breath: Circulatory/Breathing; GIT: Gastro Intestinal; PA: Pains/Aches; Bolded cells indicate highest factor loading for each symptom and hence best allocation to relevant component.

### 3.3. Prevalence and Number of Symptoms/Health Complaints Reported in Last 12 Months

Table 3 shows that the Egyptian sample had significantly higher frequency than the UK for every symptom that the study examined. Interestingly, there were similarities between the two samples in the terms of the symptoms that students reported most frequently. For instance, difficulties to concentrate, fatigue, headaches, nervousness/anxiety ranked high for both samples, followed by back pain in the UK and mood swings in Egypt.

**Table 3:** Prevalence of symptoms/health complaints during last 12 months in UK and Egypt.

Psychological	United Kingdom	Egypt	
	Sometimes/Very often	Sometimes/Very often	*p*
	N (%)	N (%)	
Depressive mood	1116 (31.1)	1732 (55.1)	<0.001
Nervousness/Anxiety	**1561 (43.3)**	**1999 (63.2)**	<0.001
Mood swings	1519 (42.1)	**2350 (74.5)**	<0.001
Difficulties to concentrate	**1959 (54.5)**	**2478 (78.1)**	<0.001
Fear/Phobia	607 (16.9)	1572 (52.6)	<0.001
Nightmares	761 (21.1)	1298 (41.4)	<0.001
Weight gain/Weight loss	1420 (39.4)	1728 (55.2)	<0.001
Lack of appetite	793 (22.2)	1438 (45.6)	<0.001
Sleep disorder/Insomnia	1223 (34.1)	2008 (63.7)	<0.001
**Circulatory/Breathing**			
Trembling hands	470 (13.1)	707 (22.7)	<0.001
Trembling	257 (7.2)	632 (20.4)	<0.001
Speech impediment	195 (5.4)	479 (15.4)	<0.001
Rapid heartbeats, Circulatory problems, Dizziness	621 (17.2)	1370 (43.6)	<0.001
Breathing difficulties	495 (13.9)	741 (23.6)	<0.001
**Gastro Intestinal**			
Diarrhoea	564 (15.7)	856 (27.4)	<0.001
Constipation	552 (15.5)	902 (28.9)	<0.001
Abdominal problems	780 (21.8)	1735 (56)	<0.001
**Pains/Aches**			
Back pain	**1552 (43.3)**	1724 (54.7)	<0.001
Neck and shoulder pain	1419 (39.4)	1630 (51.9)	<0.001
Fatigue	**2194 (61)**	**2703 (85.3)**	<0.001
Headaches	**2148 (59.6)**	**2470 (77.9)**	<0.001

All percentages are row percentages for each country rounded to one decimal point; Bolded cells indicate some of the higher frequency symptoms/health complaints during last 12 months.

In terms of the number of symptoms/health complaints reported in last 12 months (Table 4), across each of the four symptom groups, relatively more UK students than the Egyptian sample reported no symptom over the past year. Conversely, relatively more Egyptian students reported ≥4 symptoms over the past year than the UK sample.

**Table 4:** Number of symptoms/health complaints reported in last 12 months.

Psychological	Symptoms							
	United Kingdom				Egypt			
	None	1–2	3	≥4	None	1–2	3	≥4
	N (%)	N (%)	N (%)	N (%)	N (%)	N (%)	N (%)	N (%)
	604 (18)	973 (29)	443 (13.2)	1336 (39.8)	95 (3.5)	298 (10.9)	283 (10.3)	2066 (75.3)
**Circulatory/Breathing**	2215 (64.9)	1016 (29.8)	119 (3.5)	61 (1.7)	1098 (37.2)	1345 (45.5)	296 (10)	216 (7.3)
**Gastro Intestinal**	2298 (66)	1013 (29)	171 (4.9)	N/A	1037 (34.6)	1528 (60)	436 (14.5)	N/A
**Pains/Aches**	519 (14.9)	1642 (47.2)	744 (21.4)	577 (16.6)	137 (4.5)	1083 (35.8)	918 (30.3)	893 (29.5)

Psychological symptoms—9 items; Circulatory**/**Breathing—5 items; Gastro Intestinal—3 items; Pains/Aches—4 items; All symptoms counted if reported to occur Sometimes or Very often; *p* value for comparisons of number of symptoms between UK and Egypt were all significant at *p* < 0.001.

### 3.4. Frequency of Symptoms/Health Complaints by Perceived Stress Level

We explored and compared the frequency of symptoms/health complaints by perceived stress level (in Quartiles). Table 5 shows the frequency of symptoms across the four symptom groups expressed as mean rating (from 1 = “never” to 4 = “very often”) by quartiles of perceived stress. For each of the UK and Egyptian samples, across each of the four symptom groups, there was an apparent stepladder appearance whereby the frequency of symptoms increased with the increase of the level (quartile) of perceived stress. In addition, for any given stress level, Egyptian students reported a higher frequency across all symptoms than the UK sample.

**Table 5:** Frequency of symptoms by level of perceived stress for UK and Egypt.

Symptoms	UK				Egypt				P *^c^*
	Level of Perceived Stress *^a^*				Level of Perceived Stress *^b^*				
	Q1	Q2	Q3	Q4	Q1	Q2	Q3	Q4	
	M (SD)	M (SD)	M (SD)	M (SD)	M (SD)	M (SD)	M (SD)	M (SD)	
Psychological	1.76 (0.51)	2.00 (0.52)	2.16 (0.59)	2.50 (0.61)	2.45 (0.61)	2.60 (0.58)	2.76 (0.56)	2.94 (0.54)	<0.001
Circulatory/Breathing	1.28 (0.40)	1.38 (0.44)	1.49 (0.52)	1.66 (0.60)	1.72 (0.60)	1.83 (0.64)	1.89 (0.65)	2.03 (0.69)	<0.001
Gastro Intestinal	1.51 (0.58)	1.66 (0.63)	1.76 (0.67)	1.92 (0.72)	2.11 (0.70)	2.13 (0.69)	2.22 (0.69)	2.31 (0.69)	<0.001
Pains/Aches	2.23 (0.63)	2.39 (0.62)	2.49 (0.67)	2.74 (0.65)	2.69 (0.64)	2.77 (0.64)	2.90 (0.60)	2.98 (0.60)	<0.001

*^a^* UK sample, for intragroup comparison for each row, differences for values of each row were significant at *p* < 0.001; *^b^* Egyptian sample, for intragroup comparison for each row, differences for values of each row were significant at *p* < 0.001; *^c^* Denotes *p* value for intergroup comparison between the UK and Egypt; Q: quartile (Q4 = highest level of perceived stress); symptoms measured as scale between 1 to 4 (1 = “never”; 4 = “very often”); all *p*-values based on ANOVA tests.

### 3.5. Correlations between Symptoms and Perceived Stress among UK and Egyptian University Students

In order to explore the strength of the associations between the symptom groups and perceived stress for each of the two samples, an initial correlation analysis was undertaken (Table 6). There were significant positive correlations between each of the four symptom groups and perceived stress. In addition, for both countries, the highest correlation was between the psychological symptoms and perceived stress. An interesting finding was that, for all the four symptom groups, the correlations between them and stress were all higher for the UK than for Egypt.

**Table 6:** Correlations between symptoms and perceived stress among UK and Egyptian university students.

Symptom	Perceived Stress	
	United Kingdom (N)	Egypt (N)
Psychological	0.449 (3604)	0.306 (3184)
Circulatory/Breathing	0.268 (3600)	0.174 (3150)
Gastro intestinal	0.243 (3600)	0.110 (3152)
Pains/Aches	0.285 (3598)	0.196 (3182)

Pearson Correlation; Analysis did not control for any variables; All correlations significant at *p* < 0.0001.

### 3.6. Associations between Symptoms and Perceived Stress, Controlling for Sex and Age, Presented for Each Country Separately, and Combined

We then undertook linear regression analyses between the symptom groups with perceived stress as the outcome, controlling for each of the other symptom groups and also for age and sex (Table 7). In the UK, stress was associated with psychological symptoms, circulatory/breathing symptoms and pain & aches symptoms; other symptoms were not significant when controlling for (in the presence of) these three symptom groups. The higher the perceived stress the higher was the level of psychological, circulatory/breathing, and pain & aches symptoms. For Egypt, again stress was significantly associated with psychological symptoms; but females were significantly more likely to report stress than males (*p* < 0.001), and younger students were also more prone to stress than older students at the 10% significance level (*p* = 0.063). The age-sex interaction was not significant, indicating that generally, younger students were more prone to perceived stress.

Interesting results emerged when both countries were analysed together. Generally, perceived stress was highly and significantly associated with psychological symptoms in both countries alike. Age and gender (main effect) were also associated with perceived stress. However, in the UK, overall, students were significantly less stressed than Egypt, but within each country (interaction effect), women were significantly more prone to stress. Other interactions were not significant.

As the symptoms and other independent variables were highly associated, hence we further undertook stepwise regression in order to account for multicolinearity. Table 8 shows that generally, stress was highly and significantly associated with psychological symptoms and with pain & aches symptoms in both countries. UK students were less stressed than their counterparts in Egypt. Age and gender were generally also associated with stress; the younger the student was the more likely to suffer from stress. Interactions were not significant.

**Table 7:** Coefficients of Predictors of perceived stress among university students in the UK, Egypt and both countries together.

Variable	United Kingdom		Egypt		United Kingdom and Egypt	
	β	*p*	β	*p*	β	*p*
Psychological Symptoms	**0.411**	**0.000**	**0.265**	**0.000**	**0.369**	**0.000**
Circulatory/Breathing Symptoms	**0.041**	**0.027**	0.019	0.360	0.020	0.161
Gastro intestinal Symptoms	0.018	0.330	−0.024	0.217	0.000	0.984
Pains/Aches Symptoms	**0.040**	**0.034**	0.038	0.090	**0.039**	**0.006**
Age	−0.027	0.077	−0.033	0.063	**−** **0.025**	**0.040**
Sex (female)	0.016	0.303	**0.089**	**0.000**	**0.068**	**0.000**
Country (UK)	—	—	—	—	**−** **0.087**	**0.000**
Country (UK)-sex interaction	—	—	—	—	−0.036	0.137

Analysis of individual countries controlled for sex and age; Analysis of both countries combined controlled for sex, age, country, and sex-country interaction; β **=** Standardized Coefficient; Bolded cells indicate statistical significance (at least *p* < 0.05).

**Table 8:** Significant predictors of perceived stress among university students in the UK and Egypt together.

Variable	Perceived Stress	
	β	*p*
Psychological Symptoms	**0.378**	**0.000**
Country (UK)	**−** **0.112**	**0.000**
Sex (female)	**0.052**	**0.000**
Pains/Aches Symptoms	**0.042**	**0.002**
Age	**−** **0.029**	**0.019**

Bolded cells indicate statistical significance.

## 4. Discussion

As for the first objective of the study, we described and compared the general characteristics of the two samples; and undertook factor analysis of 21 self-reported symptoms/health complaints into appropriate components. Few other studies examined such a wide range of symptoms and factor analysed them into components. For both countries, our factor analysis generated a four-factor solution: psychological; circulatory/breathing; gastrointestinal; and, pains/aches symptom groups. In addition, the factor loadings of the individual symptoms onto the corresponding components were quite similar for the UK and Egypt. These findings are in support of research in Germany, where a 16-symptom list similar to that we employed was also condensed into four matching groups of symptoms in a sample comprising German students [44]. Our findings also agree with earlier research [45]. A point to note is the “convergence” in the factor analysis findings across the UK and Egypt which suggested that the symptoms list we employed performed comparably in assessing the variety of self-reported health complaints across university students with western (European) and also Middle Eastern/Eastern Mediterranean (Egyptian) cultural backgrounds.

In terms of objective two, the study assessed and compared the prevalence of 21 symptoms/health complaints, and the number of symptoms/health complaints reported in the last 12 months. The Egyptian sample reported significantly higher frequency than the UK for every symptom that was examined. In addition, there were similarities between the two samples as regards the symptoms reported most frequently: difficulties to concentrate, fatigue, headaches, nervousness/anxiety ranked high for both samples, followed by back pain in the UK and mood swings in Egypt. Our findings are in partial agreement with research across seven European countries [39] where nervousness, headache, back ache, and neck/shoulder pain were the complaints students reported most often. We are also in support of the literature, where psychological health complaints and pains/aches symptoms were generally prevalent among university students e.g., among U.S. and Canadian medical students where levels of overall psychological distress were consistently higher than in the general population [8]; and also among Middle Eastern countries e.g., Saudi Arabian students where 34% and 41% reported impaired concentration and memory disturbances respectively [9]. As for the number of symptoms/health complaints during last 12 months (Table 4), across each of the four symptom groups, relatively more UK students reported no symptom over the past year than the Egyptian sample; and conversely, relatively more students from Egypt reported ≥ 4 symptoms over the past year than the UK sample. These findings are consistent with others, where research among university students of several European countries noted that among European students, students from Turkey and Spain exhibited the highest prevalences of complaints when compared with e.g., Germany and Denmark [39]. Whilst there could be a true higher prevalence of symptoms in Egypt than in the UK, and these differences might actually reflect real differences in the prevalence of complaints, such findings might also suggest that symptoms may be commonly more often reported in Mediterranean countries as compared to central, Eastern or Northern European nations. Culture affects all aspects of health and illness, including the perception of it, the explanations for it, and the behavioral options to promote health or alleviate suffering [46]. Hence, it remains to be discovered whether/if cultural factors contribute to a higher “readiness” to report symptoms/health complaints in these countries. Methodological discrepancies might also be the cause, as research among university students e.g., measuring their stress levels and comparing the findings with results from other countries have also raised the possibility that differences could be either due to the different instruments employed in different studies [25]. In order to eliminate such possibility, in the current study, we consciously employed the same instruments across both countries. Nevertheless, such clustering of symptoms is important, and has been reported among college students in Korea [47]. This proposes that having multiple symptoms could be common among university students, where such potential “clustering of symptoms” might be due to the tendency for specific healthy (and/or unhealthy) lifestyle factors to also aggregate in clusters in university students [48,49,50].

In connection with objective three, the study explored and compared the frequency of symptoms/health complaints by perceived stress level. For any given stress level, the Egyptian students had a higher frequency across all symptoms than the UK sample, and the potential reasons behind such differences have been highlighted above. Importantly, for each of the UK and Egyptian samples, across each of the four symptom groups, there was an apparent stepladder (dose-response) appearance whereby the frequency of symptoms increased with the increase of the level (quartile) of perceived stress. Our findings are in agreement with Korea, where nursing students with higher perceived stress were significantly more likely to experience gastro intestinal symptoms [47]. However, whilst Lee and coworkers [47] examined only gastro intestinal symptoms and the stepladder pattern seemed present but less evident than ours; we examined a much wider variety (four groups) of symptoms, and the step ladder appearance was very distinct across increasing levels of stress across all the four symptom groups.

As regards objective four, we compared the association between symptoms and perceived stress without and with controlling for sex, age and country. Before controlling (correlation analysis), we found significant positive correlations between each of the four symptom groups and perceived stress; and for both countries, the highest correlation was between the psychological symptoms and perceived stress. However, whilst the frequency of symptoms (prevalences) were generally more in Egypt than in the UK (discussed above), an interesting finding was that the correlations between reported symptoms and stress were all higher for the UK than for Egypt for all the four symptom groups.

Nevertheless, when we subsequently undertook linear regression analysis between the symptom groups with perceived stress as the outcome (controlled for each of the other symptom groups and for age and sex). For both countries, gastro intestinal symptoms lost its initial association with stress; and for Egypt only, pains/aches lost its initial association with stress. Such analysis highlighted the importance of controlling for other symptoms when analysing the relationship between any given symptom group and stress, as such controlling is not often undertaken in published studies. A notable point is that, in the UK, overall, students were significantly less stressed than Egypt (discussed above), but within each country (interaction effect), women were significantly more prone to stress. This latter finding is in agreement with other research that females generally are likely to complain of stress. In Saudi Arabia, the prevalence of stress was higher among females (75.7%) than among males (57%) (odds ratio = 2.3, *p* < 0.0001) [25]; and a study of stress and health-promoting features in Australian, New Zealander, and Chilean dental students found that overall females were significantly more stressed than males [51].

We then took the analysis further, as the symptoms and other independent variables were highly associated. Hence we conducted stepwise regression in order to account for multicolinearity. The findings suggested that stress was highly and significantly associated with psychological symptoms and with pain & aches symptoms in both countries; UK students were less stressed than their counterparts in Egypt; gender (females) was generally also associated with stress (discussed above); and age was generally also associated with stress where younger students were more likely to suffer from stress.

It is generally not straightforward to pinpoint the reasons behind the observed UK-Egypt differences. In addition to the cultural aspects of the people and methodological variations of the studies noted above, other factors might include university level characteristics or even features of the country. As for the university level characteristics, understanding the different dimensions of the universities’ “fine-grain” variables necessitates further consideration, specifically as these aspects relate to determinants of student health/well- being. Few multi-level studies collected sufficient student- and university-level information to be able to further our vision of such relationships [28,50,52]. In terms of country-level variations, e.g., its political and social welfare stances, for instance, research observed that the type of welfare state regime accounted for about half of the national-level variation of health inequalities between European countries [53]. Individuals in nations with Scandinavian and Anglo-Saxon welfare (e.g., UK) regimes had better self-perceived general health as compared to Southern and East European welfare regimes [53]. Many factors can contribute to the higher stress levels observed in Egypt. For instance age, where we noted that students of the Egyptian sample (mean age 18.9 ± 1.4 years) were generally younger that their UK counterparts (mean age 24.9 ± 8.6 years) reflecting the fact that a significant fraction of students at university in Egypt are “fresh” from high school, and hence are relatively younger. Higher stress could also be related to the prevailing sociocultural atmosphere: whilst the UK environment is characterized having relatively more “freedom” for students, the Egyptian environment might be characterized by relatively more restrictions that do not exist for the UK counterparts. For instance, students at higher education institutions in Egypt are generally banned from travel overseas in the latter years of study at university and not allowed to travel aboard until they graduate and also complete their compulsory military service, a point that many students find frustrating. Another feature to consider is the type and amount of support students receive at university, where in the UK there are relatively more stress mitigating programs at universities, whilst in Egypt, stress mitigating/reducing programs are an uncommon feature of campus health services. In addition, Table 1 showed that the prevalences of smoking, illicit drug/s use, and alcohol consumption was higher among the UK students than their Egyptian counterparts, suggesting that these lifestyle factors may act as coping mechanisms to “dilute” the stress levels of the UK sample.

The current study has limitations. It is a cross-sectional study (no causal relationships can be derived). Hence while stress could be a consequence of health complaints, the relationship could also work in the opposite direction, where symptoms and health complaints build up the stress levels of students. Thus more longitudinal research is required to unpack these relationships and ascertain the time line of the links. Self-reporting was employed to approximate the frequency of symptoms; no clinical validations were conducted. Nonetheless, no valid external measurement of health complaints exists since physicians’ ratings also heavily depend on patients’ descriptions. In addition, in quantitative analysis, the HBSC symptom checklist—a list akin to that which we employed—showed satisfactory reliability [54], with test–retest reliabilities from 0.70 to 0.80. Finally, our samples remain convenience samples, thus generalizations need to be cautious. Selection bias whereby students with frequent health complaints may be less probable to be on campus during the data collection cannot be ruled out. Thus the reported prevalence of symptoms in the current study may underestimate the true burden of morbidity among these students. Despite these limitations, the study has important strengths as to the best of our knowledge, no previous study seems to have investigated in detail and compared the relationships between perceived stress and a wide range of self-reported health complaints across large samples of students from many different faculties in the United Kingdom and Egypt.

## 5. Conclusions

Across both countries, the findings suggest the need for a three pronged approach: understanding of the sources of stress; how college students experience stress; and, the coping mechanisms that different students employ to mitigate stress. In terms of the first point, educational institutions should be aware how/if their student selection practices and pre-requisites, courses, exams and course assessment methods, as well as the social and/or living environments of the university might contribute to students’ stress, in order to tailor interventions aimed at preventing, treating and caring for students’ distress. For the second point, the relationships between stress and psychosomatic health complaints need to be further unpacked, with future research on larger, more diverse student samples to better understand the prevalence and contributors of health complaints. For the third point, for instance, Hungarian nursing students with high stress and more frequent psychosomatic symptoms used inadaptive ways of coping more often; and used less effectively coping ways that aimed at problem solving and maintaining their own health [6]. Hence, the careful consideration of effective preventive/intervention strategies to help minimize the impact of stressful situations are required e.g., mindfulness-based stress reduction [55], cognitive-behavioral stress management, cardiovascular fitness, and generalized physical activity [56], or positive use of leisure time [57]. Other interventions could include self-hypnosis, meditation, feedback on various health habits, educational discussions, changes in the length and type of curriculum, and changes in the grading system [58]. Interventions that address both physical and psychological complaints may be beneficial for this population.

## References

[1] Rubin L.E. (2008). Student mental health in a chiropractic university setting. J. Chiropr. Educ..

[2] Kadison R. (2004). The mental health crisis: What colleges must do. Chron. Higher Educ..

[3] Lima M.C., Domingues Mde S., Cerqueira A.T. (2006). Prevalence and risk factors of common mental disorders among medical students. Rev. Saude Publica.

[4] Chan G.C., Kohm D. (2007). Understanding the psychosocial and physical work environment in a Singapore medical school. Singapore Med. J..

[5] Moos R.H., Van Dort B. (1979). Student physical symptoms and the social climate of college living groups. Am. J. Community Psychol..

[6] Pikó B., Piczil M. (2012). Study of stress, coping and psychosomatic health among baccalaureate nurses-to-be. Orv. Hetil..

[7] Okamoto M., Tan F., Suyama A., Okada H., Miyamoto T., Kishimoto T. (2000). The characteristics of fatigue symptoms and their association with the life style and the health status in school children. J. Epidemiol..

[8] Dyrbye L.N., Thomas M.R., Shanafelt T.D. (2006). Systematic review of depression, anxiety, and other indicators of psychological distress among U.S. and Canadian medical students. Acad. Med..

[9] Khan M.M. (2008). Adverse effects of excessive mobile phone use. Int. J. Occup. Med. Environ. Health.

[10] Chrzanowska D., Wdowiak L., Bojar I. (2004). The origin of stress, its causes, symptoms and frequency of appearance among the students of Medical University of Lublin. Ann. Univ. Mariae Curie Sklodowska Med..

[11] Bruce I.N., Harland R.W., McBride N.A., MacMahon J. (1993). Trends in the prevalence of asthma and dyspnoea in first year university students, 1972–89. Q. J. Med..

[12] Bruls V.E., Bastiaenen C.H., de Bie R.A. (2013). Non-traumatic arm, neck and shoulder complaints: Prevalence, course and prognosis in a Dutch university population. BMC Musculoskelet. Disord..

[13] Obembe A.O., Johnson O.E., Tanimowo T.O., Onigbinde A.T., Emechete A.A. (2013). Musculoskeletal pain among undergraduate laptop users in a Nigerian University. J. Back Musculoskelet Rehabil..

[14] Hayes M.J., Smith D.R., Cockrell D. (2009). Prevalence and correlates of musculoskeletal disorders among Australian dental hygiene students. Int. J. Dent. Hyg..

[15] Heuscher Z., Gilkey D.P., Peel J.L., Kennedy C.A. (2010). The association of self-reported backpack use and backpack weight with low back pain among college students. J. Manipulative Physiol. Ther..

[16] Hamilton A.G., Jacobs K., Orsmond G. (2005). The prevalence of computer-related musculoskeletal complaints in female college students. Work.

[17] Shen L., Kong H., Hou X. (2009). Prevalence of irritable bowel syndrome and its relationship with psychological stress status in Chinese university students. J. Gastroenterol. Hepatol..

[18] Kim Y.J., Ban D.J. (2005). Prevalence of irritable bowel syndrome, influence of lifestyle factors and bowel habits in Korean college students. Int. J. Nurs. Stud..

[19] Norton G.R., Norton P.J., Asmundson G.J., Thompson L.A., Larsen D.K. (1999). Neurotic butterflies in my stomach: The role of anxiety, anxiety sensitivity and depression in functional gastrointestinal disorders. J. Psychosom. Res..

[20] Hazlett-Stevens H., Craske M.G., Mayer E.A., Chang L., Naliboff B.D. (2003). Prevalence of irritable bowel syndrome among university students: The roles of worry, neuroticism, anxiety sensitivity and visceral anxiety. J. Psychosom. Res..

[21] Tan Y.M., Goh K.L., Muhidayah R., Ooi C.L., Salem O. (2003). Prevalence of irritable bowel syndrome in young adult Malaysians: A survey among medical students. J. Gastroenterol. Hepatol..

[22] Cho C.Y., Hwang I.S., Chen C.C. (2003). The association between psychological distress and musculoskeletal symptoms experienced by Chinese high school students. J. Orthop. Sports Phys. Ther..

[23] Terre L., Ghiselli W. (1995). Do somatic complaints mask negative affect in youth?. J. Am. Coll. Health.

[24] Stock C., Kücük N., Miseviciene I., Guillén-Grima F., Petkeviciene J., Aguinaga-Ontoso I., Krämer A. (2003). Differences in health complaints among university students from three European countries. Prev. Med..

[25] Abdulghani H.M., AlKanhal A.A., Mahmoud E.S., Ponnamperuma G.G., Alfaris E.A. (2011). Stress and its effects on medical students: A cross-sectional study at a college of medicine in Saudi Arabia. J. Health Popul. Nutr..

[26] Abdul Karim S., Eikemo T.A., Bambra C. (2010). Welfare state regimes and population health: Integrating the East Asian welfare states. Health Policy.

[27] El Ansari W., Maxwell A.E., Mikolajczyk R.T., Stock C., Naydenova V., Krämer A. (2007). Promoting public health: Benefits and challenges of a Europeanwide research consortium on student health. Cent. Eur. J. Public Health.

[28] El Ansari W., Clausen S.V., Mabhala A., Stock C. (2010). How do I look? Body image perceptions among university students from England and Denmark. Int. J. Environ. Res. Publ. Health.

[29] El Ansari W., Stock C., Phillips C., Mabhala A., Stoate M., Adetunji H., Deeny P., John J., Davies S., Parke S. (2011). Does the association between depressive symptomatology and physical activity depend on body image perception? A survey of students from seven universities in the UK. Int. J. Environ. Res. Public Health.

[30] El Ansari W., Labeeb S., Kotb S., Yousafzai M.T., El-Houfey A., Stock C. (2012). Correlates of smoking, quit attempts and attitudes towards total smoking bans at university: Findings from eleven faculties in Egypt. Asian Pac. J. Cancer Prev..

[31] El Ansari W., Labeeb S., Moseley L., Kotb S., El-Houfey A. (2013). Physical and psychological well-being of university students: Survey of eleven faculties in Egypt. Int. J. Prev. Med..

[32] El Ansari W., Sebena R., Stock C. (2013). Socio-demographic correlates of six indicators of alcohol consumption: Survey findings of students across seven universities in England, Wales and Northern Ireland. Arch. Public Health.

[33] El Ansari W., Stock C., Mills C. (2013). Is alcohol consumption associated with poor academic achievement in university students?. Int. J. Prev. Med..

[34] El Ansari W., Sebena R., Stock C. (2014). Do importance of religious faith and healthy lifestyle modify the relationships between depressive symptoms and four indicators of alcohol consumption? A survey of students across seven universities in England, wales, and Northern Ireland. Subst. Use Misuse.

[35] El Ansari W., Dibba E., Labeeb S., Stock C. (2014). Body image concern and its correlates among male and female undergraduate students at Assuit University in Egypt. Glob. J. Health Sci..

[36] El Ansari W., Khalil K., Crone D., Stock C. (2014). Physical activity and gender differences: Correlates of compliance with recommended levels of five forms of physical activity among students at nine universities in Libya. Cent. Eur. J. Public Health.

[37] El Ansari W., Stock C. (2012). Factors associated with smoking, quit attempts and attitudes towards total smoking bans at university: A survey of seven universities in England, Wales and Northern Ireland. Asian Pac. J. Cancer Prev..

[38] Cohen S., Kamarck T., Mermelstein R. (1983). A global measure of perceived stress. J. Health Soc. Behav..

[39] Stock C., Mikolajczyk R.T., Bilir N., Petkeviciene J., Naydenova V., Dudziak U., Marin-Fernandez B., El Ansari W. (2008). Gender differences in health complaints among students: Results from a survey in seven countries. J. Public Health.

[40] Potthoff P., Schroeder E., Reis U., Klamert A. (1999). Process and results of field work concerning the Federal Health Survey. Gesundheitswesen.

[41] American College Health Association (2006). American College Health Association-National College Health Assessment (ACHA-NCHA) spring 2005 reference group data report (Abridged). J. Am. Coll. Health.

[42] World Health Organization (WHO) (2000). Obesity: Preventing and Managing the Global Epidemic.

[43] Bruusgard D., Nessioy I., Rutle O., Furuseth K., Natvig B. (1993). Measuring functional status in a population survey. The Dartmouth COOP/WONCA functional health assessment charts used in an epidemiological study. Fam. Pract..

[44] Stock C., Krämer A. (2001). Health of students during their course of studies. Das Gesundheitswesen.

[45] El Ansari W., Oskrochi R., Stock C. (2013). Symptoms and health complaints and their association with perceived stress: Students from seven universities in England, Wales and Northern Ireland. J. Public Health.

[46] Saint Arnault D. (2009). Cultural determinants of help seeking: A model for research and practice. Res. Theory Nurs. Pract..

[47] Lee E.Y., Mun M.S., Lee S.H., Cho H.S. (2011). Perceived stress and gastrointestinal symptoms in nursing students in Korea: A cross-sectional survey. BMC Nurs..

[48] Dodd L.J., Al-Nakeeb Y., Nevill A., Forshaw M.J. (2010). Lifestyle risk factors of students: A cluster analytical approach. Prev. Med..

[49] El Ansari W., Stock C., John J., Deeny P., Phillips C., Snelgrove S., Adetunji H., Hu X., Parke S., Stoate M. (2011). Health promoting behaviours and lifestyle characteristics of students at seven universities in the UK. Cent. Eur. J. Public Health.

[50] El Ansari W., Stock C., Snelgrove S., Hu X., Parke S., Davies S., John J., Adetunji H., Stoate M., Deeny P. (2011). Feeling healthy? A survey of physical and psychological wellbeing of students from seven universities in the UK. Int. J. Environ. Res. Public Health.

[51] Gambetta-Tessini K., Mariño R., Morgan M., Evans W., Anderson V. (2013). Stress and health-promoting attributes in Australian, New Zealand, and Chilean dental students. J. Dent. Educ..

[52] Saab H., Klinger D. (2010). School differences in adolescent health and wellbeing: Findings from the Canadian Health Behaviour in School-Aged Children Study. Soc. Sci. Med..

[53] Eikemo T.A., Bambra C., Judge K., Ringdal K. (2008). Welfare state regimes and differences in self-perceived health in Europe: A multilevel analysis. Soc. Sci. Med..

[54] Haugland S., Wold B., Stevenson J., Aaroe L.E., Woynarowska B. (2001). Subjective health complaints in adolescence: A cross-national comparison of prevalence and dimensionality. Eur. J. Public Health.

[55] Song Y., Lindquist R. (2014). Effects of mindfulness-based stress reduction on depression, anxiety, stress and mindfulness in Korean nursing students. Nurse Educ. Today.

[56] Baghurst T., Kelley B.C. (2014). An examination of stress in college students over the course of a semester. Health Promot. Pract..

[57] Yarnal C., Qian X., Hustad J., Sims D. (2013). Intervention for positive use of leisure time among college students. J. Coll. Character.

[58] Shiralkar M.T., Harris T.B., Eddins-Folensbee F.F., Coverdale J.H. (2013). A systematic review of stress-management programs for medical students. Acad. Psychiatry.

